# Feasibility and Preliminary Outcomes of a Machine-Based Pilates Program on Quality of Life and Low Back Pain in Aged Adults: A Mixed-Methods Approach

**DOI:** 10.3390/medicina62061021

**Published:** 2026-05-25

**Authors:** Joana Azul, Carolina Silva, Rogério Salvador, Raúl Antunes, Pedro Duarte-Mendes, Filipe Rodrigues

**Affiliations:** 1 ESECS—Polytechnic of Leiria, 2411-901 Leiria, Portugal; joana_azul@sapo.pt (J.A.); carolinamds1234@gmail.com (C.S.); rogerio.salvador@ipleiria.pt (R.S.); raul.antunes@ipleiria.pt (R.A.); 2 Research Center in Sport, Health and Human Development (CIDESD), 5000-558 Vila Real, Portugal; 3 Department of Sports and Well-Being, Polytechnic Institute of Castelo Branco, 6000-084 Castelo Branco, Portugal; pedromendes@ipcb.pt; 4 Sport Physical Activity and Health Research & Innovation Center (SPRINT), 6000-084 Castelo Branco, Portugal

**Keywords:** quality of life, low back pain, Pilates, individualized training

## Abstract

*Background and Objectives*: The aging process impacts physical function and quality of life, often exacerbated by chronic conditions such as low back pain. This pilot study explored the feasibility and preliminary outcomes of a 12-week machine-based Classical Pilates program on quality of life and low back pain in older adults. *Materials and Methods*: An exploratory mixed-methods approach was used. The quantitative phase included 13 participants (Mage = 64.76 ± 4.71) to evaluate quality of life. All 13 participants were assessed for low back pain and quality of life, and 4 females participated in a focus group for a qualitative analysis of perceived benefits. The intervention consisted of two individual sessions per week. *Results*: Preliminary quantitative analysis revealed an increase in the physical domain of quality of life (*p* < 0.05). There was a reduction in pain intensity and global pain values (*p* < 0.05). Qualitative data provided context for these preliminary findings, with participants reporting perceived improvements in mobility, body awareness, pain management, and sleep quality, alongside notable psychosocial benefits. *Conclusions*: Preliminary findings suggest that a 12-week individual machine-based Pilates program is feasible and may be associated with improvements in the physical domain of quality of life and perceived low back pain in active older adults. Due to the complete absence of a control group and the very small sample size, it is impossible to isolate the intervention’s efficacy from natural progression or placebo effects. Consequently, these results are strictly exploratory and hypothesis-generating. The mixed-methods approach highlights that individualized machine-based Pilates may provide self-reported psychosocial and daily living benefits, supporting the need for future well-powered randomized controlled trials.

## 1. Introduction

The rapid growth of the aging population represents one of the most significant demographic shifts of the 21st century, imposing complex challenges on global healthcare systems and socio-economic structures [1,2]. In response to this demographic transition, the paradigm of “active aging” has emerged as a public health priority. As defined by the World Health Organization, active aging goes beyond simply prolonging lifespan; it emphasizes the optimization of opportunities for health, participation, and security to enhance the quality of life as people age [3]. However, the physiological process of aging is inevitably accompanied by functional declines, including a progressive loss of muscle mass, reduced joint mobility, and diminished postural control [4,5].

When these age-related declines intersect with chronic musculoskeletal conditions, the ability to maintain an active lifestyle may be severely compromised [5,6]. Among older adults, chronic low back pain is one of the most prevalent and debilitating conditions [7,8]. Epidemiological data suggest that the adult population is highly susceptible to low back pain due to degenerative spinal changes, decreased bone density, and body composition alterations. Specifically, recent epidemiological evidence highlights that obesity-related anthropometric indices, such as increased body mass index and central fat distribution, may be significant risk factors for chronic low back pain [9]. Chronic low back pain, defined as pain persisting for more than 12 weeks, extends far beyond physical discomfort. It creates a cascade of negative effects that negatively impact overall quality of life. As noted by Hadi et al. [10], chronic low back pain is strongly associated with difficulties in performing activities of daily living, sleep disturbances, kinesiophobia (fear of movement), and a heightened risk of psychological morbidities such as depression and social isolation.

To mitigate the functional decline and manage low back pain, conservative and non-pharmacological interventions are universally recommended as the first line of treatment [6]. Structured physical exercise, in particular, has proven highly effective in maintaining strength, balance, and flexibility [11]. Within this context, the Pilates method has received increasing attention. Developed by Joseph Pilates in the 1920s, this mind–body exercise modality focuses on core stability, flexibility, deep breathing, and precise movement control [12].

Current scientific evidence strongly supports the efficacy of Pilates for managing low back pain and improving quality of life. For instance, Valenza et al. [13] and Cruz-Díaz et al. [14] demonstrated that Pilates interventions significantly reduce pain intensity and functional disability in adults with chronic non-specific low back pain. Furthermore, research by Pereira et al. [15] highlights that Pilates effectively improves multiple physical domains in the elderly, offering a holistic approach to healthy aging. The method achieves these results by training the deep abdominal and lumbar multifidus muscles in a neutral lumbopelvic position, thereby reducing mechanical load on the spine and optimizing joint stability [16].

Despite these positive findings, the existing literature presents several critical limitations that warrant further investigation. First, the majority of research has focused on younger or middle-aged populations [13,14], leaving a gap in understanding how older adults, who often present with multiple comorbidities, respond to the method. Second, most studies utilize mat-based group classes or Contemporary Pilates adaptations [17]. Studies investigating Classical machine-based Pilates (utilizing equipment like the Reformer or Cadillac with adjustable spring resistance) in an individualized setting remain scarce. Machine-based Pilates may offer benefits for older adults, as the equipment provides support for fragile movements while allowing for safely progressively overloaded resistance [14,18]. Finally, the previous literature relies almost exclusively on quantitative assessments [13,14,15,16,17]. While some tools provide valuable standardized metrics, they often fail to capture the nuanced, lived experiences of the participants. A mixed-methods approach is crucial in geriatrics, as it combines measurable outcomes with the contextual insights of qualitative data (e.g., focus groups). Therefore, to address these gaps, the primary objective of this pilot study was to explore the feasibility and preliminary outcomes of a 12-week individualized, machine-based Classical Pilates program on quality of life and low back pain in active older adults. Utilizing an exploratory mixed-methods design, this study hypothesizes that the intervention may be associated with improvements in the physical domains of quality of life and reductions in perceived low back pain intensity, while fostering enhanced body awareness and psychosocial well-being as reported by the participants.

## 2. Materials and Methods

### 2.1. Design and Participants

This research was designed as an exploratory, quasi-experimental, single-arm pilot study employing a mixed-methods approach. Given the individualized nature of the Classical Pilates intervention on machines and the lack of pre-existing data on this specific protocol in older adults, a pilot design without a control group was deemed appropriate to assess feasibility and gather preliminary data. This design lays the groundwork for future large-scale randomized trials.

To ensure the safety and homogeneity of the sample, the following inclusion criteria were applied: (a) individuals aged between 55 and 75 years; (b) physically active in their daily routines; (c) no previous experience with machine-based Pilates; and (d) presenting with moderate disability (Oswestry Disability Index = 21% to 40%) caused by low back pain. Participants were excluded if they presented any of the following: (a) absolute contraindications to physical exercise; (b) severe spinal pathologies (e.g., hernias requiring surgery, severe osteoporosis, idiopathic scoliosis); (c) previous or scheduled spinal surgeries; (d) severe disability (Oswestry Disability Index > 41%); (d) chronic obstructive pulmonary disease; (e) recent stroke or myocardial infarction; (f) uncontrolled hypertension; (g) active oncological treatment; (h) or physical therapy treatment for low back pain within the six months prior to the study.

The study comprised a total sample of 13 active older adults (12 females and 1 male) aged between 58 and 72 years (M = 64.76 ± 4.71). Regarding general health and lifestyle characteristics, the majority of participants (92.3%) reported sleeping between five and eight hours per night. Chronic conditions were prevalent, with 38.5% reporting elevated cholesterol levels and 23.1% presenting with hypertension. Furthermore, 61.5% routinely took medication for individual health conditions, 76.9% had undergone previous surgeries, and all participants were physically active prior to the intervention.

The sample size was intentionally kept small due to the high logistical and financial demands of providing 24 fully individualized, one-on-one machine-based sessions supervised by a highly specialized instructor.

### 2.2. Procedures

The study was conducted in accordance with the ethical guidelines of the Declaration of Helsinki. The research protocol, including all procedures for data collection and data privacy, was reviewed and approved by the Ethics Committee of the Polytechnic Institute of Leiria (Protocol Approval Number CE/IPLEIRIA/62/2023). Although the study received prior ethical approval, it was not prospectively registered in a public clinical trial registry. As this was initially conceived as a small-scale, quasi-experimental, single-arm pilot study primarily focused on assessing feasibility and preliminary outcomes, prospective registration was not deemed strictly necessary by the research team at the time of inception. However, to ensure full methodological transparency and adhere to current editorial standards, the study has been retrospectively registered at ClinicalTrials.gov (Identifier: NCT07596602). The data collection process was divided into five phases. In the first phase, initial contact was established with local gymnasiums equipped with a specialized Pilates studio to present the study objectives. Following institutional approval (i.e., phase two), the recruitment process commenced via email invitations to local entities and verbal dissemination during group classes at the facility. In the third phase, potential participants were screened against the inclusion and exclusion criteria. Eligible individuals were informed about the study’s anonymous and voluntary nature, ensuring they understood they could withdraw at any time without negative repercussions.

During the fourth phase (baseline), participants signed the written informed consent form and completed the initial assessments, which included the sociodemographic questionnaire and the quality of life and low back pain instruments. Following the baseline assessment, the 12-week intervention was applied. Finally, in the fifth phase (post-intervention), the questionnaires were re-administered to the 13 participants, and the qualitative focus group was created (*n* = 4).

### 2.3. Instruments

To gather a complete dataset, a combination of sociodemographic, quantitative, and qualitative instruments was utilized. A sociodemographic and clinical questionnaire was developed for this study to collect baseline data regarding age, biological sex, daily sitting time, diagnosed pathologies, physical limitations, perceived pain, medication use, and exercise habits. Next participants completed the Oswestry Disability Index Version 2.0 validated to Portuguese [19]. It consists of 10 items assessing pain intensity, personal care, lifting, walking, sitting, standing, sleeping, sex life, social life, and traveling. Each item is scored from 0 (no disability) to 5 (maximum disability). Participants also completed the World Health Organization Quality of Life Brief Portuguese version [20] was used to assess subjective quality of life. It comprises 26 items divided into four distinct domains: physical, psychological, social relationships, and environment. Responses are recorded on a five-point scale, where higher scores indicate a better perceived quality of life.

To complement the quantitative findings, a semi-structured online focus group was conducted with a four-participant subsample. The interview explored pre-study physical activity habits, perceived physical benefits, improvements in low back pain, impacts on daily living, psychosocial benefits, overall satisfaction, and intentions for future lifestyle changes. To ensure anonymity, participants were coded as Participant 1 through Participant 4.

### 2.4. Intervention

A structured exercise protocol utilizing the Classical Pilates method on machines was implemented in a one-on-one setting. Previous literature highlights that individualized Pilates sessions allow for a level of control, immediate postural correction, and safety that group classes often cannot provide, which is particularly crucial for older adults presenting with pain or mobility limitations [12,18]. The intervention followed the FITT principles [21]. The program spanned 12 weeks, consisting of two individual sessions per week, with each session lasting approximately 50 min, resulting in a total of 24 individualized Pilates sessions per participant. All sessions took place in person at a partner gymnasium’s Pilates studio and were supervised by a professional with formal academic training in physical exercise and specialized certification in Classical Pilates.

The pedagogical structure of the sessions was divided into two distinct components, characteristic of the Classical approach: the “Skeleton” and the “System” [22]. The Skeleton component comprised the core base exercises performed primarily on the Reformer and the Mat. The System component involved specific adaptations and fragments of the Skeleton exercises, executed on auxiliary equipment. By utilizing the System, the instructor prepared the participants to execute the full Skeleton movements more safely and efficiently. The auxiliary equipment utilized for the System included the Wunda Chair, Cadillac, High Barrel, Electric Chair, Small Barrel, Magic Circle, elastic bands, and 500 g dumbbells. The use of machine-based Pilates, which employs adjustable spring resistance rather than body weight alone or gravity, has been shown to be highly effective in providing both necessary support for fragile movements and progressive overload for strengthening the lumbopelvic region [14,16]. Specific progression criteria were standardized across all participants. An individual progressed to a higher spring resistance or a more complex exercise variation only when they demonstrated: (a) full biomechanical control of the movement, (b) absence of self-reported pain during execution, and (c) the ability to maintain a neutral lumbopelvic position throughout the prescribed range of motion. If these criteria were not met, the participant remained at the current level or regressed to a highly supported System modification.

The goal of the 12-week program was for participants to achieve independence and proficiency in a full, basic-level Skeleton sequence on the Mat and Reformer. Given that some standard movements were overly complex or potentially unsafe for beginners experiencing low back pain, the most challenging exercises were initially omitted from the sequence [18]. Training variables were modified every two sessions (equating to one specific workout plan per week). To facilitate safe progression, the three System exercises performed at the end of each session were strategically selected to physically prepare the participants for the new Skeleton exercises that would be introduced in the following week’s sessions.

Periodization across the 12 weeks was structured to vary positional stimulus. During the first six weeks, sessions commenced with exercises on the Mat, followed by exercises on the Reformer. In the final six weeks, this order was inverted, beginning with the Reformer and concluding on the Mat. Regarding volume and load, each Skeleton exercise was performed for a single set. The prescribed number of repetitions for each specific exercise remained constant from the first to the last session of the 12 weeks. Progression and resistance modifications were achieved exclusively by altering the number of springs on the equipment, a core advantage of machine-based Pilates that facilitates highly individualized progression without altering the biomechanics of the exercise [16]. Rest periods between exercises were minimized, limited to the time required to adjust the springs or transition between machines.

Exercise intensity was continuously monitored throughout the sessions using the Talk Test [23]. This simple, non-invasive tool evaluates physical exertion based on the individual’s ability to speak and breathe comfortably during exercise. The target intensity was managed to remain light-to-moderate, ensuring that participants could maintain a fluid, comfortable conversation without exhibiting signs of severe physiological distress. This intensity management is aligned with best practices for older adults, aiming to maximize neuromuscular adaptations while minimizing cardiovascular and joint stress [21]. While objective measures like heart rate monitors provide precise cardiovascular data, the Talk Test was selected as a pragmatic, ACSM-endorsed tool for this specific demographic. Because Classical Pilates is primarily a neuromuscular and motor-control intervention rather than a high-intensity aerobic one, the focus is on breathing mechanics and precision. Furthermore, since 61.5% of the sample routinely took medications, which can often alter or blunt physiological heart rate responses (e.g., beta-blockers), the Talk Test provided a functional and reliable safety threshold for exertion. Session schedules were tailored to the availability of both the instructor and the participants, resulting in a 100% attendance rate with no missed sessions.

To ensure reproducibility and transparency, the specific 12-week exercise progression is detailed in Table 1. The intervention was periodized into two six-week blocks to vary the positional stimulus. In Phase 1 (Weeks 1–6), sessions commenced on the Mat and progressed to the Reformer. In Phase 2 (Weeks 7–12), this order was inverted. The ‘Skeleton’ exercises were progressively introduced over the weeks (e.g., adding “Rolling like a ball” exercise in Week 4, or shifting ‘The Hundred’ to the Reformer in Week 7) as participants developed core stability and motor control. The “System” exercises varied weekly and were specifically tailored using auxiliary equipment (i.e., Wunda Chair, Cadillac, Barrels) to address individual limitations and prepare the participants for upcoming Skeleton progressions.

### 2.5. Statistical Analysis

Quantitative data were exported and analyzed using the IBM Statistical Package for the Social Sciences (SPSS) Statistics, version 29. Participants with an adherence rate below 75% or who missed ten consecutive sessions were excluded from the analysis. Interestingly, adherence remained at 100% for the final sample. Descriptive statistics were calculated for all variables. The assumption of normality was confirmed using the Shapiro–Wilk test which is appropriate for samples smaller than 50. Additionally, normality was accepted as skewness and kurtosis values fell within the acceptable ranges of −2 to +2 and −7 to +7, respectively [24]. To determine the differences between the pre- and post-intervention moments, paired-sample *t*-tests were applied. The significance level was set at 5% (*p* < 0.05). Additionally, 95% Confidence Intervals (95% CI) of the mean differences were calculated to provide a measure of precision for the estimated effects. Cohen’s d was calculated to determine the magnitude of the effect sizes, interpreted as trivial (0 to 0.19), small (0.20 to 0.49), medium (0.50 to 0.79), and large (0.80 or higher) [24].

For the qualitative component, the focus group audio recording was transcribed verbatim. The data were then analyzed by two independent researchers using thematic analysis, following the guidelines proposed by Braun and Clarke [25]. The researchers familiarized themselves with the data, highlighted key expressions related to the research questions, and iteratively grouped these into broader themes and subthemes. To ensure inter-coder reliability and reflexivity, the two researchers initially coded the transcript independently. Following this independent phase, they compared their respective coding frameworks. Any discrepancies in theme generation or quote allocation were resolved through collaborative discussion until a 100% consensus was reached. A third independent senior researcher was available to adjudicate unresolved disagreements, though this was ultimately not required.

## 3. Results

**Figure 1:**
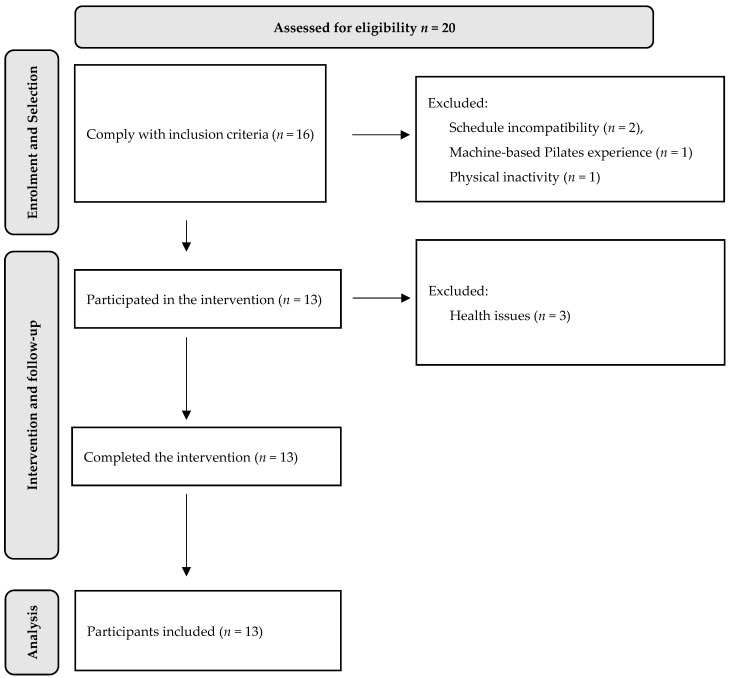
Flowchart.

The flow chart representing participant allocation is illustrated in Figure 1. Initially, from a total of 20 potential participants, 16 met the inclusion criteria and were selected to begin the intervention. The four excluded individuals did not participate due to schedule incompatibility (*n* = 2), prior experience with machine-based Pilates (*n* = 1), and physical inactivity (*n* = 1). Of the 16 participants who initiated the sessions, three withdrew early from the program due to health issues entirely unrelated to the intervention (i.e., one pre-existing injury, one daily-life fracture, and one scheduled surgery). No emergencies, injuries, or hospitalizations related to the physical exercise sessions were reported during the intervention. The final sample consisted of 13 participants, and four of these individuals subsequently participated in the qualitative focus group. Table 2 displays the pre- and post-intervention outcomes regarding low back pain disability. Statistically significant reductions (*p* < 0.05) were observed in pain intensity, discomfort while sitting, limitations in social life, and the global disability score.

Table 2 displays the pre- and post-intervention outcomes regarding low back pain disability. Statistically significant reductions (*p* < 0.05) were found in pain intensity, discomfort while sitting, limitations in social life, and the global disability score. The magnitude of the effects for these significant variables was large, ranging from 0.96 to 1.79.

Table 3 presents the descriptive and inferential statistics regarding the quality of life indicators before and after the 12-week intervention. The physical domain of quality of life was the only variable to exhibit statistically significant differences (*p* = 0.027), showing an increase in physical perception from baseline to post-intervention. The calculated Cohen’s *d* was 0.59, indicating a medium effect size. No statistically significant differences were observed in the psychological, social relationships, environment, or overall quality of life domains.

The thematic analysis of the focus group (*n* = 4) provided contextual depth to the quantitative findings, revealing overarching themes regarding program satisfaction, physical and psychosocial benefits, health literacy, and lifestyle modifications. Table 4 summarizes the primary themes, subthemes, and illustrative quotes translated from the participants’ native language.

## 4. Discussion

The present quasi-experimental and exploratory pilot study aimed to analyze the preliminary effects of a 12-week machine-based Classical Pilates program on quality of life and chronic low back pain in a sample of older adults. By adopting a mixed-methods methodology, it was possible to cross-reference quantitative trends with the qualitative perceptions of the participants. While the absence of a control group precludes conclusions about efficacy, the preliminary results support the study’s exploratory hypotheses, suggesting an association between the intervention and improvements in the physical domain of quality of life and a reduction in disability associated with low back pain.

Regarding quality of life, a statistically significant increase was observed in the physical domain. This finding aligns with recent literature, which highlights the Pilates method as a potentially valuable approach for improving functional capacity [15]. Evidence suggests that machine-based training facilitates neuromuscular adaptations that may translate into better perceived physical health and autonomy [14,26].

Concerning low back pain, despite the very small sample size for this specific analysis (*n* = 5), the quantitative data demonstrated a preliminary reduction in pain intensity, discomfort while sitting, limitations in social life, and the global disability score. These observations are consistent with previous larger-scale research. For example, Valenza et al. [13] and Cruz-Díaz et al. [14,27] reported improvements in pain and disability following structured Pilates interventions. Similarly, studies by Bhadauria and Gurudut [28] and Patti et al. [29] demonstrated significant reductions in low back pain using the Oswestry Disability Index after 12 to 14 weeks of Pilates. Conversely, da Luz et al. [17] found no significant differences in pain intensity after a shorter six-week intervention. This contrast suggests that neuromuscular adaptations in older adults occur more gradually, making a 12-week timeframe more appropriate for achieving meaningful physiological changes.

The integration of the qualitative data provides essential context for understanding the mechanisms underlying these preliminary physical improvements. During the focus group, participants explicitly reported substantial improvements in their ability to perform activities of daily living, such as sitting, standing, and walking. Furthermore, participants highlighted a profound increase in body awareness. The ability to transfer postural learning from the sessions to their daily routines, such as applying specific spine stretches before bed to alleviate tension, provides a coherent explanation for the improved quantitative pain scores and self-management capabilities. The quantitative analysis revealed a significant improvement in the “social life” variable of the low back pain assessment. This finding is supported by Villarreal-Angeles et al. [30], who demonstrated psychological and social improvements in older adults through Pilates. The qualitative data strongly reinforced this outcome. Participants described the sessions not only as physical therapy but as a psychological outlet, emphasizing the importance of the affective bonds formed with the instructors. In an aging population where social isolation is prevalent, the supportive environment of individualized training proved to be highly beneficial. Additionally, although the standardized questionnaires did not detect statistically significant improvements in sleep quality or the broader psychological domain of quality of life, the qualitative reports consistently emphasized more restorative sleep and clear mental health benefits. This discrepancy between quantitative and qualitative data is a common phenomenon in exploratory studies, where closed-ended instruments may lack the sensitivity to capture subtle subjective experiences in the short term [10].

The absence of statistically significant results in the psychological, social, and environmental domains of the quality of life questionnaire can be attributed to several factors. The 12-week duration may be insufficient to promote quantifiable changes in these broader domains, as studies reporting widespread psychosocial benefits often span six months or more [31]. Furthermore, participants had a high baseline level of physical activity, which may attenuate the magnitude of the observed effects compared to the improvements frequently reported in previously sedentary populations [32].

### Limitations and Future Directions

The results of this exploratory study must be interpreted with extreme caution due to several critical methodological limitations. The primary weakness is the single-arm, quasi-experimental design lacking a control group. Consequently, it is impossible to infer a direct causal relationship between the Pilates program and the observed improvements, as outcomes could be influenced by external factors, the placebo effect, the natural progression of the participants’ conditions, or the positive psychological impact of the social interaction inherent in one-on-one sessions. Secondly, the overall sample size is very small (*n* = 13). Although all 13 participants were included in the quantitative statistical analyses for both quality of life and low back pain, with a smaller subgroup of *n* = 4 participating in the qualitative focus group, a sample of 13 still limits statistical power, the stability of the estimates, and the generalizability of the findings. As with any early-stage pilot design, this limits statistical power, the stability of the estimates, and the generalizability of the findings. Consequently, the large effect sizes observed in our subsample should be interpreted with caution, as small samples are prone to overestimating these metrics. This renders the quantitative pain outcomes strictly preliminary and hypothesis-generating. Furthermore, external confounding variables, such as the frequency of analgesic medication use and participation in other physical activities during the study, were not rigorously controlled. Thirdly, as noted regarding reproducibility, the exclusive reliance on the Talk Test to monitor exercise intensity is a subjective limitation. While pragmatic for this clinical population, it does not guarantee consistent or reproducible physiological training intensity across all participants. Future trials should incorporate objective tools, such as heart rate telemetry or the rating of perceived exertion (e.g., Borg Scale), to precisely quantify internal training loads Fourthly, our inclusion criteria deliberately selected older adults who were already physically active. Consequently, these preliminary findings cannot be generalized to frail, sedentary, or functionally impaired elderly populations, who might exhibit entirely different physiological adaptations or require substantially modified, lower-intensity training protocols. Although this clinical variability reflects the ecological reality and typical health profile of the aging population, thus enhancing the external validity of the preliminary findings, we acknowledge that it introduces potential confounding variables that limit the internal validity of the study. Future randomized controlled trials should employ larger samples to allow for statistical stratification based on specific comorbidities and medication profiles. Fifthly, the sample was predominantly female (i.e., 12 out of 13 participants). While this skewed sex distribution may reflect the real-world demographic trend of Pilates practitioners, it constitutes a sex bias. Consequently, these preliminary findings cannot be generalized to older male populations, as neuromuscular adaptations, pain perception, and psychosocial responses to the intervention may differ significantly by sex. Future trials must employ targeted recruitment strategies to ensure a balanced sex representation and allow for sex-stratified comparative analyses. Sixthly, the qualitative component included only four participants. While sufficient for gathering preliminary contextual insights in a pilot study, this small sample size precludes the achievement of true data saturation. Consequently, the qualitative themes cannot be considered exhaustive, and future studies should employ larger qualitative cohorts or multiple focus groups to ensure thematic saturation and a more comprehensive phenomenological understanding.

To address these significant gaps, future research must utilize well-powered, randomized controlled trials with larger, representative samples. Investigating the dose–response effect through long-term interventions and directly comparing machine-based Classical Pilates with mat-based interventions or usual care is essential. Despite these limitations, this pilot study demonstrates the feasibility of the intervention, evidenced by 100% adherence, and provides valuable qualitative insights that justify further scientific inquiry into machine-based Pilates for geriatrics.

## 5. Conclusions

The findings of this exploratory pilot study suggest that a 12-week individualized, machine-based Classical Pilates program is highly feasible and may be associated with positive trends in both the physical domain of quality of life and the management of chronic low back pain in active older adults. Quantitatively, the intervention was associated with preliminary improvements in physical health perception and self-reported reductions in pain intensity and global disability. However, due to the small sample size and the lack of a control group, these findings cannot be interpreted as definitive evidence of efficacy. The integration of qualitative data highlighted the holistic value of this approach, with participants reporting subjective improvements in sleep hygiene, mobility, and a heightened sense of body awareness. The psychosocial benefits and strong social connections formed during the individualized sessions underscore the potential of this method to support mental well-being. Ultimately, we emphasize that these findings are exploratory and hypothesis-generating. They warrant the design of robust, well-powered randomized controlled trials to isolate the efficacy of individualized machine-based Pilates from placebo effects or natural condition progression in active aging.

## Figures and Tables

**Table 1:** Overview of the standardized classical Pilates protocol.

Component	Modality	Phase 1: Weeks 1–6 (Start on Mat)	Phase 2: Weeks 7–12 (Start on Reformer)
Skeleton	Mat	Base sequence (reps): The Hundred (100), Half Roll Back (5), [progressed to Roll Up in W2], One Leg Circle (5), Single Leg Stretch (10), Double Leg Stretch (10), Scissors (10), Spine Stretch Forward (5).	Maintained full sequence from W6. (Note: Mat sequence performed at the end of the session in this phase).
Skeleton	Reformer	Footwork: Toes (10), Arches (10), Heels (10). Stomach Massage: Hands Back (10), Round (10), Reaching (4) added in W3. Short Box: Flat (5), Side to Side (3), Front Stretch (3), Tree (3) added in W4, Hug/Round (5) added in W5. Long Stretch: One Leg Press (10), Elephant (10) added in W2. Knee Stretch: Round (8), Arched (8) added in W6.	Footwork: Tendon Stretch (10) added in W9. Added to Reformer: The Hundred (100) [W7], Pelvic Lift (10) [W7], Running (10) [W8], Leg Circles & Frog (5) [W10], Knee Stretch—Knees Off (10) [W10].
System	Auxiliary Equipment	Weekly variations to support Mat/Reformer: Examples include: Half Roll Back Bar, Push Through, Front Ballet Stretches, Breathing (Cadillac), Push Down, Pumping Parallel (Wunda Chair), Small Barrel extensions.	Advanced supportive variations: Examples include: Leg/Arm Springs, Hanging Pull Up, Monkey, First part of Teaser (Cadillac), Mermaid, Pull Up, Knee Curls (Wunda Chair), Elastic Band work.

**Table 2:** Descriptive and inferential statistics for low back pain disability indicators.

Variable	Pre		Post		*t*	*p*	d	95% CI (Diff)
	M	SD	M	SD				
Pain intensity	1.40	0.55	0.60	0.89	2.14	<0.05	0.96	−0.24, 1.84
Personal care	0.20	0.45	0.00	0.00	1.00	0.19	-	−0.36, 0.76
Lifting	3.00	0.00	2.00	1.58	1.41	0.12	-	−0.96, 2.96
Walking	0.20	0.45	0.00	0.00	1.00	0.18	-	−0.36, 0.76
Sitting	1.60	0.89	0.80	0.84	2.14	<0.05	0.96	−0.24, 1.84
Standing	1.80	1.30	1.40	1.52	1.633	0.89	-	−0.28, 1.08
Sleeping	1.20	0.45	1.00	1.22	0.53	0.31	-	−0.84, 1.24
Sexual life *	-	-	-	-	-	-		-
Social life	1.40	0.89	0.60	1.34	4.00	<0.05	1.79	0.24, 1.36
Traveling	1.00	0.00	1.20	1.30	−0.34	0.37	-	−1.82, 1.42
Global value	27.60	9.07	17.70	13.02	3.97	<0.05	1.78	2.98, 16.82

Notes: SD = Standard Deviation; *t* = *t*-test value; *p* = significance value at 0.05; d = effect size magnitude; 95% CI (Diff) = 95% Confidence Interval of the mean difference. * The “Sexual life” item was not applicable to this sample and was excluded from the analysis.

**Table 3:** Descriptive and inferential statistics for quality of life indicators.

Variable	Pre		Post		*t*	*p*	d	95% CI (Diff)
	M	SD	M	SD				
Physical domain	27.69	3.95	28.92	3.73	−2.13	0.027	0.59	−2.49, 0.03
Psychological domain	23.31	2.29	23.85	2.94	−1.17	0.133	-	−1.54, 0.47
Social relationships	11.90	0.99	11.90	1.10	0.00	0.500	-	−0.58, 0.58
Environment	30.18	3.37	31.18	3.03	−1.70	0.060	-	−2.31, 0.31
Overall quality of life	7.84	1.62	7.85	1.46	0.00	0.500	-	−0.43, 0.43

Notes: SD = Standard Deviation; *t* = *t*-test value; *p* = significance value at 0.05; d = effect size magnitude; 95% CI (Diff) = 95% Confidence Interval of the mean difference.

**Table 4:** Qualitative thematic analysis of the focus group regarding the Pilates intervention.

Main Theme	Subtheme	Illustrative Quote	*n*
Project Satisfaction	Overall satisfaction	“*It is indeed very good because it is highly individualized work, very well monitored; every movement is technically guided.*” (P1)	4
Challenges	Less positive aspects	“*Difficulty in the exercises, some harder than others, but everything was overcome. The only real difficulty is the economic one, I mean, maintaining exercises of this kind financially.*” (P3)	4
Physical Benefits	General physical outcomes	“*When I left there, I slept better. For example, during the day, walking used to hurt, and now when I move around, I practically feel nothing.*” (P2)	4
	Low back pain reduction	“*I have a sciatica problem and gradually my mobility improved, the way I move has improved.*” (P4)	4
Psychosocial Benefits	Mental health and social connection	“*I felt that the moment there was almost like being at the psychologist; it is very pleasant to disconnect from real-life problems during that period, it felt very good. Besides, I also maintained a connection… I managed to form an affective bond with them which helped me a lot.*” (P1)	4
Knowledge and Literacy	Body awareness	“*That help, that awareness of our body, of our breathing… “low the shoulder before we do something, raise the shoulders, put the shoulder in place”… all of that echoes in our heads and we learn to do things correctly.*” (P4)	4
Lifestyle Changes	Intentions to remain active	“*If I had the means, I would do these two sessions a week, or even just one session a week, because it is truly worth it.*” (P2)	4
	Daily habit integration	“*Those spine stretching positions that are great for people with back pain, I take the opportunity to do them at night before going to bed to see if it brings relief, especially when I am more tense. These are things that stick with us and we then apply them in our daily lives.*” (P2)	4
Program Importance	Individual and societal value	“*I think the importance of these projects is precisely to alert people our age and not only, younger ones too, to a physical exercise that is different from the usual and that helps us in our lives, our well-being, our mental and physical health. It would be very good if there were more projects like this!*” (P1)	4
Professionalism	Instructor competence	“*I think either of the two people who accompanied me demonstrated a lot of professionalism. And in a delicate way, the way they managed to correct something I was doing less well.*” (P1)	4

Notes: Quotes were translated into English; (P#) denotes the randomized participant identifier to maintain anonymity.

## Data Availability

The data presented in this study is available on request from the corresponding author. The data is not publicly available due to privacy and ethical restrictions regarding participant anonymity.
